# Genome-Wide Association Study for Lactation Performance in the Early and Peak Stages of Lactation in Holstein Dairy Cows

**DOI:** 10.3390/ani12121541

**Published:** 2022-06-14

**Authors:** Mahsa Zare, Hadi Atashi, Miel Hostens

**Affiliations:** 1 Department of Animal Science, Shiraz University, Shiraz 7144113131, Iran; mahsa.zare91@yahoo.com (M.Z.); atashi@shirazu.ac.ir (H.A.); 2 Department of Population Health Sciences, University of Utrecht, Yalelaan 7, 3584 CL Utrecht, The Netherlands

**Keywords:** milk yield, lactation stage, genome-wide association study, Holstein dairy cow

## Abstract

**Simple Summary:**

Although genome-wide association studies (GWAS) have been carried out within a variety of cattle breeds, they are mainly based on the accumulated 305-day lactation yield traits estimated by summing the test-day recorded every day during the lactation period, or combining the weekly or monthly test-day records by linear interpolation. Since the additive genetic variance for milk yield and composition changes during lactation, the genetic effects of QTL related to these traits are not constant during the lactation period. Therefore, a better understanding of the genetic architecture of milk production traits in different lactation stages (e.g., beginning, peak, and end stages of lactation) is needed. The aim of this study was to detect genomic loci associated with lactation performance during 9 to 50 days in milk (DIM) in Holstein dairy cows. Candidate genes identified for milk production traits showed contrasting results between the EARLY and PEAK stages of lactation. Based on the results of this study, it can be concluded that in any genomic study it should be taken into account that the genetic effects of genes related to the lactation performance are not constant during the lactation period.

**Abstract:**

This study aimed to detect genomic loci associated with the lactation performance during 9 to 50 days in milk (DIM) in Holstein dairy cows. Daily milk yield (MY), fat yield (FY), and protein yield (PY) during 9 to 50 DIM were recorded on 134 multiparous Holstein dairy cows distributed in four research herds. Fat- and protein-corrected milk (FPCM), fat-corrected milk (FCM), and energy-corrected milk (ECM) were predicted. The records collected during 9 to 25 DIM were put into the early stage of lactation (EARLY) and those collected during 26 to 50 DIM were put into the peak stage of lactation (PEAK). Then, the mean of traits in each cow included in each lactation stage (EARLY and PEAK) were estimated and used as phenotypic observations for the genome-wide association study. The included animals were genotyped with the Illumina BovineHD Genotyping BeadChip (Illumina Inc., San Diego, CA, USA) for a total of 777,962 single nucleotide polymorphisms (SNPs). After quality control, 585,109 variants were analyzed using GEMMA software in a mixed linear model. Although there was no SNP associated with traits included at the 5% genome-wide significance threshold, 18 SNPs were identified to be associated with milk yield and composition at the suggestive genome-wide significance threshold. Candidate genes identified for milk production traits showed contrasting results between the EARLY and PEAK stages of lactation. This suggests that differential sets of candidate genes underlie the phenotypic expression of the considered traits in the EARLY and PEAK stages of lactation. Although further functional studies are needed to validate our findings in independent populations, it can be concluded that in any genomic study it should be taken into account that the genetic effects of genes related to the lactation performance are not constant during the lactation period.

## 1. Introduction

Milk performance traits are among complex traits affecting profitability in dairy cattle. These traits are influenced by management strategies, environmental conditions, physiological stages, and genetic merit of the animal [1,2]. Over the last decades, advances in genome sequencing technologies and availability of a huge number of genetic variants in the form of single nucleotide polymorphisms (SNP) make it possible to identify genome regions underlying complex traits such as lactation performance in dairy cattle [3,4]. Although genome-wide association studies (GWAS) carried out on a variety of cattle breeds identified many genomic regions and candidate genes associated with milk production traits, they are mainly based on the estimated breeding value, daughter yield deviation, and deregressed proof for the 305-day lactation yield traits [5,6,7,8,9]. The 305-day milk yield is estimated by summing the test-day recorded every day during the lactation period, or combining the monthly test-day records by linear interpolation [10]. It has been well documented that the additive genetic variance for milk yield and composition changes during lactation [11,12,13]; therefore, the genetic effects of genomic regions associated with these traits are not constant during the lactation period. Hence, many genomic regions whose genetic effects change during the lactation might not be detected in this approach [1,14]. Therefore, a better understanding of the genetic architecture of milk production traits in different lactation stages (e.g., beginning, peak, and end stages of the lactation) is needed. Previous studies indicated that heritability in the peak of lactation is higher than in other lactation phases; therefore, a comparison of the main genomic regions associated with the EARLY and PEAK stages of lactation would promote useful information about the genetic architecture of milk production traits [13,15]. The aim of this study was to detect genomic loci associated with lactation performance during 9 to 50 days in milk (DIM) in Holstein dairy cows.

## 2. Materials and Methods

### Animals and Phenotype

The used dataset consisted of daily milk yield (MY), fat yield (FY), protein yield (PY), fat percentage (FP), and protein percentage (PP) recorded during 9 to 50 days in milk (DIM) on 134 multiparous Holstein dairy cows in 4 research herds (Aarhus University, Aarhus, Denmark; UCD Lyons Research farm, University College Dublin, Ireland; Agri-Food and Biosciences Institute, Belfast, UK; and Leibniz Institute for Farm Animal Biology, Dummerstorf, Germany). The diet, fed as a total mixed ration (TMR), consisted of corn silage, alfalfa hay, barley grain, fat powder, beet pulp, and feed additives. The included animals were milked two times daily (morning and evening). Daily milk yield was estimated as the sum of the morning and evening milk yields. The milk sample used for measuring fat and protein contents was composed of 50% morning milk and 50% evening milk. Milk samples were analyzed for composition of protein and fat content by mid-infrared analysis (Foss, Hillerød, Denmark). Fat- and protein-corrected milk (FPCM) yield was predicted as: FPCM kg=MY kg ×(0.337+0.116 FP%+0.06× PP%). Energy-corrected milk (ECM) yield was predicted as: ECM kg=MY kg ×(0.25+0.122×FP%+0.077×PP%) [16]. Fat-corrected milk (FCM) yield was predicted as: FCM kg=0.40×MY kg+15×FY kg [17]. The records collected (predicted) during 9 to 25 DIM were put into the early stage of lactation (EARLY) and those collected during 26 to 50 DIM were put into the peak stage of lactation (PEAK). Then, means of traits in each cow included in each lactation stage (EARLY and PEAK) were estimated and used as phenotypic observations for the genome-wide association study.

## 3. Genome-Wide Association Study

The included animals were genotyped using the Illumina BovineHD Genotyping BeadChip (Illumina Inc., San Diego, CA, USA) for more than 777,000 single nucleotide polymorphisms (SNP). Quality control for SNPs was performed using PLINK [18]. Non-mapped SNPs, and those with minor allele frequency (MAF) less than 5% were excluded. SNPs with call rate less than 95%, or a Hardy–Weinberg equilibrium *p* value less than 6.4 × 10^−8^ were excluded. In total, 585,109 SNPs were kept for the analyses. Genome-wide association studies (GWAS) were performed as described by Tetens et al. [19] using a linear mixed model approach through GEMMA [20]. The centered relatedness matrix was created using all SNPs. The association test was then performed with phenotype, genotype, and the centered relatedness matrix files. Univariate linear mixed models were used, and each SNP was fitted as a covariate. The likelihood ratio test was used for each SNP against the null hypothesis of g = 0 using the following statistical model.

(1) y=Wα+xβ+u+ε

where **y** is a n × 1 vector of phenotype values for the individuals (milk yield and composition); **W** is an n × c matrix of the included fixed effect (mean, herd, parity, calving year, calving season, and age at the first calving (AFC, three classes)); **α** is an c × 1 vector of the corresponding coefficients including the intercept; **x** is a vector of marker genotypes; **β** is the vector of marker effects; **u** is the vector of random polygenic effects with a covariance structure as **u**∼N(0, **K**Vg), where **K** is genomic based additive genetic relationship matrix, and Vg is the polygenic additive variance; and **ɛ** is the vector of residuals with a covariance stricter as **ɛ**∼N(0, **I**Ve), where **I** is an identity matrix and Ve is the residual variance. The Bonferroni method was used to account for multiple comparisons [21]. The thresholds of the Bonferroni corrected *p* values for suggestive and 5% genome-wide significance association were set as 1.74 × 10^−6^ (1 divided by the number of SNPs) and 8.68 × 10^−6^ (0.05 divided by the number of SNPs), respectively [22].

## 4. Gene Prospection

In a post-GWAS study, gene ontology enrichment analysis can be performed to investigate pathways and biological processes that are shared by candidate genes identified for a given trait [23]. In this study, to identify possible candidate genes associated with studied milk production traits, genes located within the 100 kb flanking regions of associated SNPs were identified using the Biomart tool [24] embedded in the Ensembl Genes database version 93 (https://www.ensembl.org/index.html, accessed on 15 October 2018). The bovine genome UMD 3.1 (https://oct2018.archive.ensembl.org/Bos_taurus/Info/Index, accessed on 15 October 2018) was used as the reference genome. The list of candidate genes identified for each trait was uploaded to Enrichr to perform gene ontology (GO) enrichment analyses [25,26]. Significantly enriched terms were identified based on the retrieved adjusted *p* value.

## 5. Results

**Figure 1:**
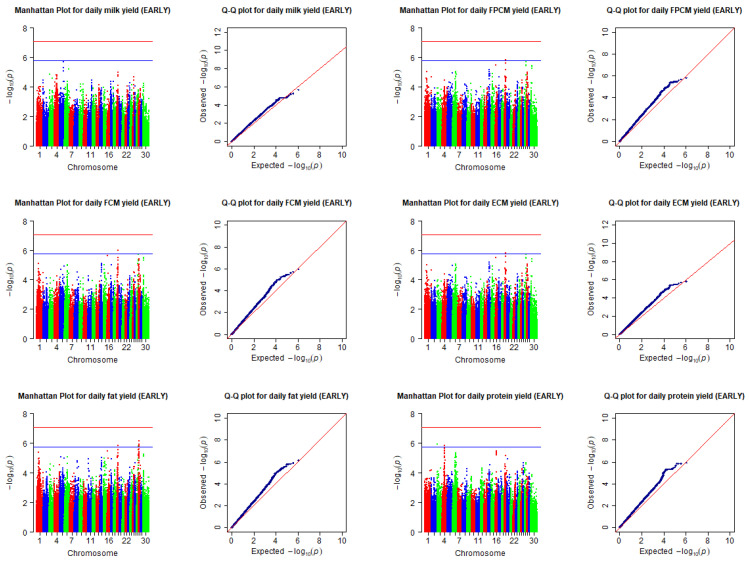
Manhattan and Q-Q plots of SNPs associated with milk yield and composition collected (predicted) for the EARLY stage of lactation. The horizontal red and blue lines in the Manhattan plots indicate the genome-wide significance threshold (−log10(8.7 × 10^−8^)), and the suggestive significance threshold (−log10(1.74 × 10^−6^)). Chromosomes are shown in red (1, 4, 7, 10, 13, 16, 19, 22, 25, and 28), blue (2, 5, 8, 11, 14, 17, 20, 23, 26, and 29), or green (3, 6, 9, 12, 15, 18, 21, 24, 27, and 30). The records of the traits collected (predicted) during 9 to 25 days in milk were put into the early stage of lactation (EARLY). FPCM = fat- and protein-corrected milk yield; FCM = fat-corrected milk yield; ECM = energy-corrected milk yield.

**Figure 2:**
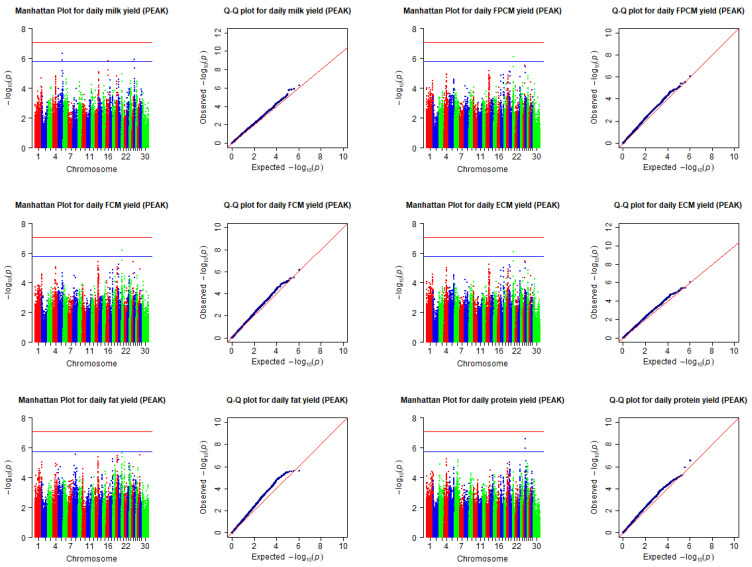
Manhattan and Q-Q plots of SNPs associated with milk yield and composition collected (predicted) for the PEAK stage of lactation. The horizontal red and blue lines in the Manhattan plots indicate the genome-wide significance threshold (−log10(8.7 × 10^−8^)), and the suggestive significance threshold (−log10(1.74 × 10^−6^)). Chromosomes are shown in red (1, 4, 7, 10, 13, 16, 19, 22, 25, and 28), blue (2, 5, 8, 11, 14, 17, 20, 23, 26, and 29), or green (3, 6, 9, 12, 15, 18, 21, 24, 27, and 30). The records of the traits collected (predicted) during 26 to 50 days in milk were put into the peak stage of lactation (PEAK). FPCM = fat- and protein-corrected milk yield; FCM = fat-corrected milk yield; ECM = energy-corrected milk yield.

Descriptive statistics for the included traits in the EARLY and PEAK stages of lactation are presented in Table 1. The cows were genotyped for a total of 777,962 SNPs, of which 585,109 passed all quality control criteria and were used for the GWAS analysis. Manhattan and Q-Q plots of SNPs associated with the considered traits in the EARLY and PEAK stages of lactation are presented, respectively, in Figure 1 and Figure 2. Although there was no SNP associated with included traits at the 5% genome-wide significance threshold, 18 SNPs were identified to be associated with milk yield and composition at the suggestive genome-wide significance threshold.

The identified SNPs, along with their position, and 100 kb flanking genes are presented in Table 2 (Assembly UMD3. 1, annotation release 103). Four SNPs (BTA5 (*n* = 2), BTA16, and BTA26) were identified to be associated with daily MY at the PEAK stage of lactation; however, no SNP was found to be associated with MY at the EARLY stage of lactation. There was one SNP on BTA21 and one SNP on BTA19 associated with daily ECM, FPCM, and FCM yields at the EARLY and the PEAK stages of lactation, respectively. Six SNPs (BTA19, BTA27, BTA28 (*n* = 4)) were identified to be associated with daily FY at the EARLY stage of lactation; however, no SNP was found to be associated with daily FY at the PEAK stage of lactation. Four SNPs (BTA3 and BTA4 (3)) and two SNPs (BTA26) were identified to be associated with PY at the EARLY and PEAK stages of lactation, respectively.

Significantly enriched biological processes enriched by candidate genes identified for considered traits are presented in Table 3. Functional genes identified as candidate genes for MY at the PEAK stage of lactation were *VWF*, *ANO2*, *XCL2*, *XCL1*, and *MYOF*, which participate in terms related to regulation of T cell chemotaxis and regulation of T cell migration. Genes including *UBB*, *PIGL*, *TRPV2*, *CENPV*, *ZMAT4*, *ZNF37A*, and *ZNF33B* were identified as candidate genes for FY at the EARLY stage of lactation. Establishment of mitochondrion localization and microtubule-mediated and regulation of the intrinsic apoptotic signaling pathway by the *p53* class mediator are among the most important terms enriched by the candidate gene identified for FY. *ATF6* and *ELMO1* were identified as candidate genes for PY at the EARLY stage, and *MYOF* was the only candidate gene identified for PY at the PEAK stage of lactation. Candidate genes identified for PY enriched terms related to regulation of transcription from the RNA polymerase II promoter in response to stress.

## 6. Discussion

Genes including *VWF*, *ANO2*, *XCL2*, *XCL1*, and *MYOF* were identified as candidate genes for MY in the PEAK stage of lactation. The association between *ANO2* and *VWF* and MY has been reported by previous studies [27,28,29]. It has been reported that an increased expression level of *VWF* is associated with increased MY in Holstein dairy cows [27]. In addition, *VWF* and *ANO2* have been reported as candidate genes for metabolic body weight in dairy cows [30]. Positive regulation of T cell chemotaxis and positive regulation of T cell migration were identified as the most important terms enriched by *VWF*, *XCL1*, and *XCL2* genes. Regulation of T cell chemotaxis and migration may have an important role on mammary gland immunity, decreasing the rate of mastitis and improving MY [31,32]. Previous studies reported that *XCL1* and *XCL2* are associated with inflammation, immunity mechanisms, and environmental adaptation in cattle [33], as well as heat tolerance in buffalo [34]. It has been reported that *MYOF* is associated with female fertility in dairy cattle [35].

We identified *WFIKKN2* and *LUC7L3* as candidate genes for FPCM, ECM, and FCM yields in the EARLY stage of lactation. The results showed that terms related to the regulation of transforming growth factor are enriched by *WFIKKN2* and *LUC7L3* genes. The *WFIKKN2* gene has been previously reported to be associated with growth and skeletal muscle [36,37], but there is no report on the effect of variation in *WFIKKN2* or *LUC7L3* on the lactation performance in cattle.

Functional genes associated with FY at the EARLY stage of lactation were *UBB*, *PIGL*, *TRPV2*, *CENPV*, *ZMAT4*, *ZNF33B*, and *ZNF37A*. Buzanskas et al. [38] reported that *ZMAT4* participates in the apoptotic, biological, developmental, and metabolic processes and is associated with reproductive performance in cattle. It has been reported that *ZMAT4* is associated with milk yield at the beginning of lactation, milking speed, and temperament in dairy cattle [39,40]. Previous studies showed that *ELMO* and *TRPV2* are associated with resistance to mastitis in dairy cattle [41,42].

Functional genes associated with PY in the EARLY stage of lactation were *ATF6* and *ELMO1*, while *MYOF* was associated with PY at the PEAK stage of lactation. Galliou et al. [43] reported that *ATF6* is associated with the number of inseminations per conception and first service conception in Holstein dairy cows. This study showed that candidate genes found for PY participate in the regulation of transcription from the RNA polymerase II promoter in response to stress, which can be considered as a mechanism to relieve the stress caused by a negative energy balance in the EARLY stage of lactation.

## 7. Conclusions

Candidate genes identified for considered traits showed contrasting results between the EARLY and PEAK stages of lactation. This suggests that differential sets of candidate genes underlie the phenotypic expression of the analyzed traits in different stages of lactation. Because few animals were used, the findings of this study need to be interpreted with caution; furthermore, further reverse transcription quantitative real-time PCR (RT-qPCR) studies are needed to validate our findings in independent populations.

## Figures and Tables

**Table 1:** Descriptive statistics for milk yield and composition in the EARLY and PEAK stages of lactation ^1^ (*n* = 137).

	EARLY		PEAK	
Trait ^2^	Mean	SD	Mean	SD
Daily milk yield (kg)	35.2	5.57	39.61	6.97
Daily FPCM yield (kg) ^3^	37.3	6.27	38.67	6.25
Daily FCM yield (kg) ^4^	37.7	6.34	39.44	6.20
Daily ECM yield (kg) ^5^	40.2	6.77	41.41	6.69
Daily fat yield (kg)	1.58	0.30	1.58	0.26
Daily protein yield (kg)	1.20	0.23	1.18	0.24
Daily fat percentage (%)	4.51	0.56	4.10	0.51
Daily protein percentage (%)	3.43	0.31	3.01	0.22

^1^ The records collected (predicted) during 9 to 25 (mean = 17 d) and 26 to 50 (mean = 38 d) days in milk were put into the EARLY and PEAK stages of lactation, respectively. ^2^ The examined traits included daily milk yield (MY), daily fat- and protein-corrected milk (FPCM), daily fat-corrected milk (FCM), energy-corrected milk (ECM), daily fat yield (FY), and protein yield (PY). ^3^ FPCM kg=MY kg ×(0.337+0.116× FP %+0.06× PP %). ^4^ FCM kg=0.4×MY kg+15×FY kg. ^5^ ECM kg=MY kg ×(0.25+0.122×FP%+0.077×PP %).

**Table 2:** Single nucleotide polymorphisms (SNP) detected by genome-wide association study (GWAS) for daily milk yield and composition collected (predicted) during EARLY and PEAK stages of lactation ^1^.

Trait	Lactation Stage	SNP ^2^	BTA ^3^	Position ^4^	*p* ^5^	100 kb Flanking Genes ^6^
Daily milk yield (kg)	EARLY	-	-	-	-	-
Daily milk yield (kg)	PEAK	BovineHD0500029986	5	104728767	1.43 × 10^−6^	*LOC100297751*, *VWF*, *ANO2*
		BovineHD0500029987	5	104731396	5.00 × 10^−7^	*LOC100297751*, *VWF*, *ANO2*
		BovineHD1600010600	16	37008841	1.48 × 10^−6^	*XCL2*, *LOC104974416*, *XCL1*
		BovineHD2600003772	26	14775291	1.24 × 10^−6^	*LOC104976766*, *MYOF*
Daily FPCM yield (kg) ^7^	EARLY	BovineHD1900010590	19	36576999	1.53 × 10^−6^	*LOC101902244*, *LOC104975057*, *LOC512899*, *WFIKKN2*, *LUC7L3*
Daily FPCM yield (kg)	PEAK	BovineHD2100004343	21	15903999	8.25 × 10^−7^	*LOC104975331*
Daily FCM yield (kg) ^8^	EARLY	BovineHD1900010590	19	36576999	1.07 × 10^−6^	*LOC101902244*, *LOC104975057*, *LOC512899*, *WFIKKN2*, *LUC7L3*
Daily FCM yield (kg)	PEAK	BovineHD2100004343	21	15903999	6.71 × 10^−7^	*LOC104975331*
Daily ECM yield (kg) ^9^	EARLY	BovineHD1900010590	19	36576999	1.57 × 10^−6^	*LOC101902244*, *LOC104975057*, *LOC512899*, *WFIKKN2*, *LUC7L3*
Daily ECM yield (kg)	PEAK	BovineHD2100004343	21	15903999	8.20 × 10^−7^	*LOC104975331*
Daily fat yield (kg)	EARLY	BovineHD1900009968	19	33866094	1.50 × 10^−6^	*LOC100296637*, *LOC101907886*, *LOC104975044*, *UBB*, *PIGL*, *TRPV2*, *CENPV*
		BovineHD2700010009	27	35283307	1.62 × 10^−6^	*LOC104976121*, *ZMAT4*
		BovineHD2800003727	28	12969171	1.58 × 10^−6^	*LOC101905153*, *LOC104970905*, *ZNF37A*, *LOC104970929*, *ZNF33B*
		BovineHD2800003728	28	12970103	1.24 × 10^−6^	*LOC101905153*, *LOC104970905*, *ZNF37A*, *LOC104970929*, *ZNF33B*
		BovineHD2800003739	28	12999108	1.51 × 10^−6^	*LOC101905153*, *LOC104970905*, *ZNF37A*, *LOC104970929*, *ZNF33B*
		BovineHD2800003747	28	13037081	6.93 × 10^−7^	*LOC101905153*, *LOC104970905*, *ZNF37A*, *LOC104970929*, *ZNF33B*
Daily fat yield (kg)	PEAK	-	-	-	-	-
Daily protein yield (kg)	EARLY	BovineHD0300002517	3	7761414	1.16 × 10^−6^	*ATF6*
		BovineHD0400016455	4	60555650	1.40 × 10^−6^	*ELMO1*
		BovineHD0400016458	4	60561187	1.40 × 10^−6^	*ELMO1*
		BovineHD0400016461	4	60566765	1.44 × 10^−6^	*ELMO1*
Daily protein yield (kg)	PEAK	BovineHD2600003771	26	14773836	1.12 × 10^−6^	*LOC104976766*, *MYOF*
		BovineHD2600003772	26	14775291	2.62 × 10^−7^	*LOC104976766*, *MYOF*

^1^ The records of the traits collected (predicted) during 9 to 25 days in milk were put into the early stage of lactation (EARLY) and those collected during 26 to 50 days in milk were put into the peak stage of lactation (PEAK). ^2^ Single nucleotide polymorphism (SNP) name. *^3^ Bos taurus* chromosome number. ^4^ Position of the SNP on the chromosome. ^5^ *P* value of the mixed model for single SNP association analysis. ^6^ Genes in 100 kb flanking regions of SNP position. Official gene symbol (Assembly UMD3.1, annotation release 103). ^7^ FPCM = fat- and protein-corrected milk yield ^8^ FCM = fat-corrected milk yield. ^9^ ECM = energy-corrected milk yield.

**Table 3:** Gene ontologies (GO) terms enriched by 100 kb flanking genes for SNPs associated with milk production traits in the EARLY and PEAK stages of lactation ^1^.

	EARLY		PEAK	
Trait	GO Term Description	Genes	GO Term Description	Genes
MY ^2^			Positive regulation of T cell chemotaxis (GO:0010820) Regulation of T cell chemotaxis (GO:0010819) Positive regulation of T cell migration (GO:2000406)	*VWF*, *XCL1*, *XCL2*
FCMY ^3^ FPCMY ^4^ ECMY ^5^	Negative regulation of cellular response to transforming growth factor beta stimulus (GO:1903845) Negative regulation of transforming growth factor beta receptor signaling pathway (GO:0030512) Transforming growth factor beta receptor signaling pathway (GO:0007179)	*WFIKKN2*, *LUC7L3*		
FY ^6^	Establishment of mitochondrion localization, microtubule mediated (GO:0034643) Regulation of intrinsic apoptotic signaling pathway by p53 class mediator (GO:1902253)	*PIGL*, *TRPV2*, *CENPV*		
PY ^7^	Positive regulation of transcription from RNA polymerase II promoter in response to stress (GO:0036003) Positive of transcription from RNA polymerase II promoter in response to endoplasmic reticulum stress (GO:1990440)	*ATF6*, *ELMO1*		

^1^ The records collected (predicted) during 9 to 25 days in milk were put into the early stage of lactation (EARLY) and those collected during 26 to 50 days in milk were put into the peak stage of lactation (PEAK). ^2^ Daily milk yield. ^3^ FPCM = daily fat- and protein-corrected milk yield. ^4^ FCM = daily fat-corrected milk yield. ^5^ ECM = daily energy-corrected milk yield. ^6^ Daily fat yield. ^7^ Daily protein yield.

## Data Availability

The data are available upon reasonable request.
